# Audit of dental record-keeping at a university dental hospital

**DOI:** 10.4102/hsag.v28i0.2442

**Published:** 2023-12-20

**Authors:** Mpule A.L. Moshaoa, Keitumetse Taunyane, Phumzile Hlongwa

**Affiliations:** 1 Department of Orthodontics, Faculty of Health Sciences, University of Pretoria, Pretoria, South Africa; 2 Department of Orthodontics, Faculty of Health Sciences, University of the Witwatersrand, Johannesburg, South Africa

**Keywords:** audit, record-keeping, clinical dental records, hospital records, record audit

## Abstract

**Background:**

Good record-keeping is fundamental in clinical practice and essential for practising dental practitioners and those in training.

**Aim:**

This study aimed to evaluate the level of compliance with clinical record-keeping by undergraduate dental students and staff at a university dental hospital.

**Setting:**

The selected study setting was the Admissions and Emergency section at a university dental hospital.

**Methods:**

A retrospective, cross-sectional review was undertaken of 257 clinical records. The CRABEL scoring system was used to evaluate 12 variables. The 12 variables included: patient name, patient hospital number, date of examination, patient main complaint, medical history, dental history, proposed treatment, proposed procedure for next visit, patient consent signature, treatment and treatment codes, student name and signature, clinical supervisor name and signature. STATA® 13 was used for descriptive analysis and all tests were conducted at 5% significance level.

**Results:**

The median CRABEL score was 87 and interquartile range (IQR: 70–92). A CRABEL score of 100 was achieved by the students in the variable *patient main complaint*, indicating a 100% compliance with this variable. Other variables such as *signature of supervisors* showed poor compliance. The CRABEL scores showed no statistically significant difference (*p* = 0.86) between the students and clinical supervisors.

**Conclusion:**

The overall audit showed that there was poor compliance with record-keeping.

**Contribution:**

The study highlights the importance of good record keepings so that key information can be accessed for proper diagnosis and treatment of the patient. An electronic filing system presents an alternative manner of documenting medical records.

## Introduction

Comprehensive and accurate patient record-keeping is an important part of good medical and dental practice. Good record-keeping is a fundamental aspect of good clinical governance and an essential competency in the training of dental students and practising dental practitioners. Accurate dental records are important to be able to deliver quality patient care. These records play a significant role in teaching and enabling research. Clinical audits allow for quality assurance to take place in order to assess and maintain the quality of care that patients receive (Kinn 1997).

Accurate dental record-keeping is a legal requirement and aids forensic investigations when they are desired (Borrman et al. 1995; Pullen & Loudon 2006). Patient records can be documented manually or electronically (Pullen & Loudon 2006) while utilising a computerised patient management system (Rothwell, Haglund & Morton 1989).

The Health Professions Council of South Africa (HPCSA) has developed guidelines on good record-keeping by medical and dental practitioners. Dental records should be kept for 6 years and, in the case of children, until the child is 25 years old. In South Africa, records in provincial hospitals and clinics should only be destroyed if authorised by the Deputy Director-General (Health Professions Council of South Africa 2019).

Medical and dental records are created by a practitioner at the time of consultation and clinical examination of a patient, as well as when documenting a medical or surgical procedure (De Klerk 1993).

Dental records usually comprise a detailed account of a patient’s personal details, medical and dental history, history of the illness, medical alerts, precautions and current treatment and medications. It also documents the clinical examination of the extra-oral and intra-oral dento-facial region, diagnosis, treatment plan, treatment procedures and management of a patient. Supplementary records may include intra-oral and extra-oral dental radiographs and clinical photographs (Charangowda 2010).

The CRABEL scoring system (an acronym from the authors CRAwford – BEresford – Lafferty), proposed in 2001, is a method for auditing medical records (Crawford, Beresford & Lafferty 2001). It ascribes a numerical score to assessments and evaluations can be made against a set of criteria based on the gold standard for medical- and surgical note-taking and record-keeping. The CRABEL scoring has four groupings: initial clerking, subsequent entries, consent and discharge letter. Appropriate boxes are marked according to the degree of compliance. Points are subtracted based on the quality of the medical notes and records. Other methods of auditing patient records that have been reported include, inter alia, the Adjusted Note Keeping and Legibility (ANKLe) score (Dexter, Hayashi & Tysome 2008) and the Surgical Tool for Auditing Records (STAR) (Tuffaha et al. 2012).

Jawaid et al. (2013) used the CRABEL scoring at Dow University and reported a 71% compliance rate. They also observed the necessity for frequent audits to enhance and sustain the quality of records, especially in the training of junior doctors (Jawaid et al. 2013). A South African study (Chamisa & Zulu 2007), conducted by the Department of Surgery at Prince Mshiyeni Memorial Hospital reported on the use of the CRABEL score to audit case notes. The study reported an 80% compliance rate for 16 out of 35 standards, and 100% was achieved for eight operation sheet standards, but a few items fell short of 80% compliance. These items were: patient’s name on every page (71%), hospital number on every page (50%), every entry timed (16%), clinician’s name printed on every note (8%), clinician’s designation on every entry (2%), an entry each weekday (77%), type of admission (9%), presenting complaint (61%), history of presenting complaint (65%), previous medical history (76%), drug history (47%), allergies (59%), social history (34%), family history (11%), each entry legible (65%) and anaesthetist’s name (69%). The recommendations from their results highlighted the importance of improved quality of note-taking as well as increased frequency of audits and the need for symposia with the medical staff team regarding the expected guidelines. Furthermore, legible and accurate note-taking should be encouraged from an undergraduate training level (Chamisa & Zulu 2007).

A study was conducted at MEDUNSA Oral Health Centre, a dental teaching hospital in Gauteng, South Africa (Mthethwa & Matjila 2019), to evaluate missing or incomplete clinical records of repeat patients who were consulted during July 2017. The study reported that 3.6% of records were fully completed, and 50% of the records were less than 80% completed (Mthethwa & Matjila 2019).

The purpose of this study was to assess the standards of record-keeping by the students and dentists at the university dental hospital using the CRABEL scoring method.

## Methods and materials

### Study design and setting

A retrospective, cross-sectional study was conducted to evaluate the compliance with dental record-keeping from 01 October 2018 to 30 September 2019. The selected study setting was the Admissions and Emergency section in a university dental hospital setting.

### Study sample

A sample size calculation, using a 95% confidence interval with a 5% margin of error, estimated that 362 clinical records were required to conduct the study for the selected study period (Raosoft 2019). The records were selected using systematic sampling, starting with a random number. Numbers from 15 to 20 were mixed in a hat and a random number was selected for sampling. In this instance, number 19 was selected and, from there, every 19th record was chosen until 362 records had been selected for evaluation.

Because of the poor filing system, 105 files could not be retrieved. Therefore, only 257 records were evaluated.

### Data collection

A scoring system for this audit was modified from the CRABEL scoring system by incorporating biographical information. The CRABEL scoring method was chosen for this study as it could easily be incorporated into the study setting when compared with other scoring methods such as ANKLe and STAR, which use clinical and surgical notes and scrutinise the legibility.

The first visit entry was examined in each set of records. The scoring system consists of 12 variables in clinical records including: patient name (5 points), patient hospital number (5 points), date of examination (5 points), patient main complaint (5 points), medical history (10 points), dental history (10 points), proposed treatment (10 points), proposed procedure for next visit (10 points), patient consent signature (10 points) treatment and treatment codes (10 points), student name and signature (10 points) and clinical supervisor (CS) name and signature (10 points). A total score of 100 points was calculated for the 12 variables, meaning that when the 12 variables were added together, they amounted to the total score of 100.

Points were deducted from the score for each entry when records were incomplete. The points deducted were standardised. For variables with a maximum of 5 points, 3 points were subtracted if information was incomplete and all 5 points were subtracted if information was missing. Similarly, for variables with a maximum of 10 points, 5 points were subtracted if information was incomplete and all 10 points were subtracted if information was missing. For example, partially completed (PC) information in each variable with a maximum of 5 points was scored 2 out of 5. Partially complete information in each variable with a maximum of 10 points was scored 5 out of 10. In cases where records were signed but the signature was illegible, 5 points were deducted for the signature variable.

The total score for CRABEL scoring system is 100. Because of the fact that the signature variable was scored 10 for students and 10 for supervisors a score of 20 points was allocated to a supervisor when they were not supervising a student so that the scoring still amounted to a CRABEL score of 100 for clinicians. Therefore, a score of 10 points was allocated to a supervisor when they were supervising a student (i.e. 10 for the student and 10 for the supervisor). This was to maintain the total CRABEL scoring of 100 for clinicians. The CRABEL score was then calculated by subtracting the total points for incomplete entries from 100 to give a final score for each record. Based on the findings of the study, recommendations were suggested.

### Data analysis and statistics

A coding sheet was developed for data collection that facilitated analysis with STATA version 14 statistical software (STATA Corp 2015). The descriptive statistics with frequency, median and interquartile range (IQR) were calculated and presented as numbers and percentages. The Wilcoxon rank-sum test and the Fisher’s exact test were used to analyse the CRABEL scores, with *p*-values of less than 0.05 considered statistically significant and 95% confidence intervals reported. One week after the first assessment, repeat measurements were conducted for 10% of the records that were randomly selected to determine intra-examiner reliability. CRABEL scores of these records were also assessed for the inter-examiner reliability.

### Ethical considerations

The Human Research Ethics Committee of the University of the Witwatersrand provided ethical approval M191133 for the study. Confidentiality and anonymity is maintained throughout the study.

## Results

The evaluated records accounted for 71% of the estimated sample, which could be considered representative of the studied population. Of the 257 records, 30 were PC with no indication of the clinician, while 15 records had been signed by student (ST), and 212 records were signed by the CS. Table 1 shows the demographic characteristics of the sample.

**TABLE 1:** Demographic characteristics of the sample.

Characteristics	Sample size (*n* = 257)	Percentage
Partially completed records	30	11.7
Student records	15	5.8
Clinical supervisor records	212	82.5
**Total**	**257**	**100**

Note: Median CRABEL = 87; Interquartile range = 70–92; Standard deviation =19.2.

The Wilcoxon rank-sum test was computed to compare the CRABEL scores between the ST and CS and showed no statistically significant difference (*p* = 0.86). Furthermore, the Fisher’s exact test was conducted (*p* ≥ 0.05), indicating that there was no statistical difference between the CRABEL scores for records taken by students and those taken by CSs.

Table 2 shows the percentage scores of the different groups (PC, ST and CS) on each of the CRABEL score variables. A CRABEL score of 100 indicates a 100% compliance with record-keeping.

**TABLE 2:** The percentages of the different groups that scored totals on the different CRABEL variables.

Variables	PC (*n* = 30) (%)	ST (*n* = 15) (%)	CS (*n* = 212) (%)
Patient name	63.4	93.3	86.8
Patient hospital number	36.7	53.4	51.4
Date of examination	53.4	80.0	82.0
Patient main complaint	56.7	100.0	97.2
Medical history	36.7	93.3	76.7
Dental history	23.3	66.7	52.9
Proposed treatment	16.7	93.3	70.8
Proposed procedure for next visit	16.7	93.3	74.0
Patient’s consent signature	56.7	80.0	94.8
Teatment and treatment codes	10.0	66.7	94.8
Student’s signature	0.0	66.7	-
Clinical supervisor’s signature	0.0	2.4	38.7

PC, partially completed; ST, signed by student; CS, clinical supervisor.

The PC’s highest score was in the *patient name* variable (63.4%), and the lowest was in both *student signature* and *clinician signature* (0%). The ST scored highest in the *patient main complaint* variable (100%) and *student signature* (66.7%), compared with the CS with 97% on *patient main complaint* and 2.4% on *clinician signature.* Full compliance overall (100%) was achieved only in the *patient main complaint* CRABEL variable by the ST, as shown in Table 2.

Inter- and intra-examiner reliability testing was conducted using 10% of the files 1 week after the initial records were audited. The inter-examiner reliability resulted in Cohen’s kappa coefficient of κ = 0.43 at 95% confidence level. According to Gisev, Bell and Chen (2013) and Landis and Koch (1977), this means that there was a moderate agreement between the examiners. The intra-examiner reliability testing resulted in κ = 0.68 at a 95% confidence level, meaning that there was substantial agreement for repeat measurements taken by PI.

## Discussion

The CRABEL score is a short, simple and reproducible means of measuring and assessing the standard of record-keeping (Rai et al. 1991). Routine audits of patient records can help to advance the standard of record-keeping. Awareness among clinicians of upholding standards in their record-keeping is important. Our study examined only 257 of the originally proposed 362 files. This was because of the illegibility of the file number recorded in the patient administration book at the Admissions and Emergency (A&E) department, which resulted in 105 irretrievable files. Of the 257 retrieved files, 30 files had no indication of the treating clinician.

The overall standard of record-keeping took into consideration the individual CRABEL variables, where ST scored an excellent 100% in recording of the main complaint, which shows that great care was taken for this variable. However, this group lacked most in recording the clinician signatures (2.4%). This may imply that students did not complete or present their files to the supervisors for their signatures, or it may simply reflect an error in supervision. With the PC group, the highest score was recorded for patient name, which is 63.4%, meaning that 36.6% of unsigned records were also devoid of patient names. For PC, the recording of the clinician signatures (0%) showed the poorest compliance. These files were PC but ‘no clinician signed’ was recorded at the end of the entry.

The HPCSA requires that the following minimum information should be available in a patient’s medical record (Health Professions Council of South Africa 2019; Medical Protection Society 2014):

- patient-specific identifiable details
- medical, dental, socioeconomic and psychological history of the patient, including allergies and habits
- the date of each consultation
- the examination of the patient’s illness
- the recommended treatment or management for the patient
- the prescribed medication and dosages
- if any, the specifics of a referrals to specialists
- any side effects from prior treatment or medication
- special investigation results (laboratory, radiology and so forth) and the patient’s indication of informed consent.

The 257 patient records were assessed using the CRABEL score and yielded results of a median score of 87 with the IQR being 70–92. This shows that the majority of the records were within the acceptable range for compliance. However, one should still strive to improve the standard to reach perfection.

A study by Ho et al. (2005) revealed that their first (of three) audits was the lowest, with greater improvement in the subsequent two audits. Myuran et al. (2017) had an electronic CRABEL score and found their results improved over 6 months with monthly audits ranging from 89% to 94%. The majority of the records were PC. They illustrated partially complete patient names, partial entry of the date, incomplete patient main complaint, medical and dental history, incomplete entries of proposed treatment, treatment and procedures of next visit. The treatment codes were not always completed, there were partial signatures, or no signatures at all. Although all 12 of the variables were computed to calculate the CRABEL score, their medians were sub-optimal.

Medical and dental history provides information pertaining to previous illnesses, experiences, diagnoses and treatment that a patient may have undergone in order for the clinician to gain a holistic understanding of the patient (Mortazavi, Rahmani & Rahmani 2015). This is critical prior to deciding which procedure can be performed safely on a specific patient (Bernoni & Leeuw 2008; American Academy of Pediatric Dentistry, Council on Clinical Affairs 2012; Salem, Villagracia & Dignah 2015). The results from this study showed that 76.7% of the records did not record medical history and that 23.3% either had PC medical history or it was completely lacking. The dental history compliance results were 52.9%, meaning that 47.1% of the records were either partially complete or totally lacking in this respect. The deficiencies seen in this variable resulted from incomplete information being recorded in the medical or dental history or sections being left completely blank. As this section may result in life-threatening circumstances (e.g. allergies and conflicting medications being administered), all clinicians should improve on this. Dental history helps to communicate to the treating clinician whether the patient has experienced dental treatment previously. The clinician is able to implement the appropriate behaviour-management techniques, ideally planning a long first appointment for first-time patients, whereas repeat patients may be given a standard appointment time.

## Limitations of the study

This was a hospital-based study. The findings of this study therefore cannot be generalised. Furthermore, the sample size was reduced owing to poor record-keeping.

## Recommendations

The following recommendations emerged from the study:

- An electronic data-capturing system is useful in the capturing and storage of data. Our study showed that a large number of files were irretrievable owing to illegibility and/or files that were lost. A study found that an electronic system for storing records is essential for consistency in noting the progress and information of a patient (Zegers et al. 2011). An electronic filing system presents an alternative manner of documenting medical records. Clinicians should be conscious of the record capturing process that should be of the highest quality as it may suggest the level of care given (Pullen & Louden 2006).
- Having immediate access to key information, such as patients’ diagnoses, allergies, laboratory test results and medications, would improve the ability of caregivers to make sound clinical decisions in a timely manner.

## Conclusion

The results from the present study showed that overall, there is a poor level of compliance with record-keeping standards. The CRABEL score is an appropriate instrument to measure compliance as it is efficient, consistent and can be duplicated in other departments.
